# Storage Conditions of Textile Dosimeters for 2D UV Dose Measurements

**DOI:** 10.3390/ma18092146

**Published:** 2025-05-07

**Authors:** Elżbieta Sąsiadek-Andrzejczak, Piotr Maras, Marek Kozicki

**Affiliations:** 1 Department of Mechanical Engineering, Informatics and Chemistry of Polymer Materials, Faculty of Materials Technologies and Textile Design, Lodz University of Technology, Żeromskiego 116, 90-543 Lodz, Poland; 2 Department of Radiotherapy Planning, Copernicus Hospital, Pabianicka 62, 93-513 Lodz, Poland; piotr.maras@wp.pl

**Keywords:** flexible dosimeter, UV radiation, technical textiles, storage conditions, surface dose

## Abstract

This paper presents the optimization of storage conditions for textile dosimeters for ultraviolet radiation measurements, which are based on cotton-woven fabric and nitroblue tetrazolium chloride (NBT) as a radiation-sensitive compound. The results of changes in light reflectance and color coordinates depending on the storage time of the samples over six months from their manufacturing under various storage conditions are presented. The results obtained for cotton—NBT dosimeters, unirradiated and irradiated with a UVC dose of 100 mJ/cm^2^, stored under the following conditions were compared: (i) at room temperature (23–25 °C, humidity 40–60%), without access to light; (ii) in a fridge (3–5 °C, humidity 70–90%), without access to light; (iii) in a freezer (−17 to −20 °C, humidity 80–90%), without access to light; and (iv) at room temperature (23–25 °C, humidity 40–60%), with access to light. Additionally, it was presented that the cotton–NBT dosimeters were suitable for 2D measurement of UV radiation doses after a period of eight months. The obtained results complement previous studies on cotton–NBT textile dosimeters and are crucial for determining the conditions of use and the expiry date of such systems.

## 1. Introduction

The storage conditions of commercially used dosimeters for measuring high-energy radiation, including ionizing and ultraviolet radiation dose measurements, are strictly defined to ensure correct and repeatable readings during control measurements and medical procedures such as radiotherapy (procedures using gamma radiation, electrons, and protons) or phototherapy (procedures using radiation of a specific wavelength of white light and ultraviolet radiation). In the case of new dosimeters, the development of conditions for repeatable manufacturing, measurement procedures, and the behavior of the dosimeter over time is essential for planning application tests and introducing the finished product to the market. In most cases, regardless of the form of the dosimeter and the type of radiation to be monitored, manufacturers recommend following several key principles that ensure the accuracy and reliability of such systems. These recommendations include rules regarding (i) storing dosimeters at a specific temperature [1]; (ii) avoiding high humidity [2]; (iii) eliminating contact with electromagnetic radiation, including ionizing and ultraviolet (UV) radiation; (iv) protection against contact with water and chemical substances; (v) separation from used dosimeters; and (vi) applying calibration procedures. It is also undesirable to bend, stretch, or expose the dosimeters to mechanical damage, including impact, shock, and vibration. In addition, UV radiation dosimeters should be stored away from white light in tightly closed packages because accidental irradiation from artificial light sources can cause erroneous readings [3,4,5,6]. In the case of reusable dosimeters, e.g., electronic or thermoluminescent, it is also important to zero them correctly after irradiation [3,4,7]. Moreover, simply following the recommendations for storing dosimeters and their expiration date does not always guarantee correct and repeatable measurement results. Often, when measuring doses and the dose distribution of high-energy radiation in 2D and 3D space, several different types of dosimeters are used to eliminate possible errors.

In the case of textile dosimeters for measuring UV and ionizing radiation, the following systems are known: (i) polyamide-2,3,5–triphenyltetrazolium chloride (PA-TTC); (ii) polyamide-nitro blue tetrazolium (PA-NBT) [8]; (iii) cotton-nitro blue tetrazolium-Pluronic F-127 (cotton-NBT-Pluronic); (iv) cotton-leuco malachite green (cotton-LMG); (v) cotton-crystal violet (cotton-LCV); and (vi) cotton-nitro blue tetrazolium (cotton-NBT) [9,10]. It has been demonstrated that cotton–NBT textile dosimeters are suitable for the 2D assessment of UV and ionizing radiation. These dosimeters can be used to measure a dose distribution of up to 10 J/cm^2^ for UV radiation and 80 kGy for ionizing radiation. For all cotton—NBT systems, basic characteristic parameters were also determined, including (i) a dynamic dose range, (ii) a linear dose range, (iii) a threshold dose, and (iv) dose sensitivity. The advantage of textile dosimeters over commercially used UV films is their greater flexibility and the possibility of placing such a dosimeter on a 3D surface during irradiation to record the dose distribution on objects of different shapes [9,10]. Dosimeters are prepared from an aqueous solution of NBT without additional chemical modifiers, by-products, or waste, which makes the production ecological. Thus, the production of cotton—NBT dosimeters is fast, cheaper, and can be carried out in larger quantities using commercially available machines designed to produce standard textile products. Once finished, they can also be used as an element of protective clothing for employees exposed to UV and ionizing radiation. By maintaining established conditions for the production, storage, and use of such dosimeters, better accessibility to such measurement systems can be ensured. Over the last fifteen years, our research group (DosLab (DosLab is a research group at the Lodz University of Technology, Lodz, Poland. Its members are co-authors of this work, as well as other radiation and polymer chemists, physicist and medical physicist, and materials engineers (https://mkozicki-sci.eu/nowa149.php, accessed on 12 March 2025))) has published results that only assessed the stability of cotton—NBT samples stored at room temperature and covered with aluminum foil for 30 days from the time of their preparation. In previous research, the influence of storage conditions on changes to textile dosimeters was not discussed.

The aim of this work was to establish the optimal storage conditions for a 2D dosimeter based on cotton fabric and an aqueous solution of NBT, produced by the padding–squeezing—drying method. To determine the changes occurring during the long-term storage of non-irradiated and 100 mJ/cm^2^ UVC-irradiated samples, the samples were kept in different temperature conditions and with or without access to daylight. The following were investigated: (i) the effect of storage conditions on the stability of non-irradiated samples within six months of preparation; (ii) the effect of storage conditions on the stability of samples irradiated with a dose of 100 mJ/cm^2^ within six months of preparation; (iii) the possibility of irradiating samples after six months of storage under different storage conditions; and (iv) measurement repeatability for non-irradiated and 100 and 10,000 mJ/cm^2^ samples within six months of preparation. To characterize the changes that occurred over time, the response of the cotton—NBT dosimeter to the UVC radiation dose at different storage stages was determined by light reflectance measurements, and the color development in the CIELAB color system was assessed based on the measurement of color coordinates. Moreover, the possibility of using cotton—NBT dosimeters stored in different conditions eight months after their preparation to measure the 2D dose distribution after non-homogeneous UVC irradiation was presented. For this purpose, the color changes of calibration samples were assessed, and the textile dosimeter was scanned using a flatbed scanner and a dedicated computer software package as a dosimetry tool. Based on the obtained data, the optimal conditions and storage time of the cotton—NBT dosimeter were determined and discussed.

## 2. Materials and Methods

### 2.1. Preparation of Cotton—NBT Dosimeter Samples

Cotton woven fabric with the following parameters was used for this study: 100% cotton; twill weave; number of warp threads: 240/dm; number of weft threads: 220/dm; thickness: 0.68 mm; mass per area: 250 g/m^2^; white bleached fabric without optical brighteners (Royal Ten-Cate, Pendergrass, GA, USA). Before modification with the UV-sensitive compound, the cotton fabric was washed to remove contaminants such as dust and residues from processing. For this purpose, a water bath containing 1.3 g/dm^3^ of a non-ionic surfactant (Rokafenol N8-P7, Boruta Zgierz, Zgierz, Poland) was used. Washing was carried out in an automatic washing machine for 30 min at 40 °C according to ISO 6330:2021 [11]. After washing, the fabric was rinsed with water and distilled water to remove the washing liquor and the surfactant residues and then dried at 40 °C for 120 min. Then, the fabric was ironed with an automatic thermal press (at 180 °C for 30 s, Blue Press Line 3S, Schulze, Berlin, Germany) to eliminate creases that could adversely affect the wetting of the samples during modification with the solution containing the radiation-sensitive compound. The fabric was padded with the solution using a batch method in two stages: padding—squeezing (E. Benz, Stuttgart, Germany) and drying (Binder FD Classic, BINDER GmbH, Tuttlingen, Germany). Cotton samples (20 × 20 cm^2^) were soaked in a 1% aqueous solution of nitroblue tetrazolium chloride (NBT, M = 817.64 g/mol, Roth, Karlsruhe, Germany) and squeezed mechanically (roller clamp 45 kg/cm, rotational speed 4 m/min). Then, the samples were dried at 30 °C for 120 min. The sample preparation process diagram is presented elsewhere [9]. In the next step, the prepared samples were cut with scissors into smaller pieces: (i) 5 × 5 cm^2^ (samples for light reflectance measurements) and (ii) 2.5 × 2.5 cm^2^ and 9 × 11 cm^2^ (samples for calibration and non-homogeneous irradiation). To determine the amount of radiation-sensitive compound deposited on the sample, the mass of the samples was measured before and after chemical modification. The mass of a 5 × 5 cm^2^ cotton fabric sample before modification in the NBT bath was 1.14 g; after squeezing, it was 1.73 g; and after drying, it was 1.27 g. After preparation, to avoid accidental irradiation, samples of cotton–NBT were wrapped in aluminum foil and stored away from radiation sources.

### 2.2. Storage Conditions

Cotton—NBT samples were packed in aluminum foil, each sample separately, to avoid direct contact during storage. To assess the influence of storage conditions on changes in dosimeters, four sample packages were prepared and stored in the following conditions: (i) at room temperature (23–25 °C, humidity 40–60%); (ii) in a fridge (3–5 °C, humidity 70–90%); and (iii) in a freezer (−17 to −20 °C, humidity 80–90%) (LG GBP31DSLZN fridge; Seoul, Republic of Korea). Temperature and humidity measurements were performed with the SBS-DL-123E electronic sensor (temperature measurement range from −40 to 125 ± 0.5 °C and humidity 0–100 ± 3%, Steinberg System, Berlin, Germany). Additionally, a package of cotton—NBT samples not covered with aluminum foil was prepared and stored at room temperature of 23–25 °C and humidity 40–60%. These samples had access to artificial light emitted by lamps illuminating the laboratory room. No additional measurements of UV radiation from these lamps were taken. Temperature changes in ambient, fridge, and freezer conditions result from (i) closing and opening the fridge and freezer, (ii) temperature fluctuations in the laboratory room during the day, and (iii) seasonal changes in the laboratory room during the experiment over an eight-month period from summer to winter.

### 2.3. UV Irradiation of Cotton—NBT Samples

Cotton—NBT samples were irradiated in a UVC chamber (Analytik Jena GmbH + Co. KG, Jena, Germany) equipped with five 8 W lamps, type G8T5, 253.7 nm (Sankyo Denki Tokyo, Japan). The distance between the UV source and the bottom of the chamber was 18 cm. The UV dose (mJ/cm^2^) was automatically delivered by the built-in control system of the device. To irradiate the cotton—NBT sample with a dose of 100 mJ/cm^2^, the exposure time was 1.8 min. To confirm that the given radiation dose was emitted by the UV chamber, an external Lutron UVC radiation detector (Lutron Instruments, Coopersburg, PA, USA) was used for the measurement. For example, for a dose of 500 mJ/cm^2^, the average of 10 measurements was 497 ± 5 mJ/cm^2^. Therefore, the doses given in the work are treated as doses emitted by the UVC chamber.

Cotton—NBT samples stored in the fridge and freezer were taken out two hours before UVC irradiation to reach ambient temperature and eliminate water vapor condensation on the sample surface. The time to heat the sample to 23 °C was approximately 35 and 55 min for the samples taken from the fridge and freezer, respectively. Temperature measurements on the sample surface were performed using a Sentry ST630 pyrometer (measuring range: −20–320 °C; measurement accuracy: ±2 °C; Sentry Optronics Corp., Taiwan, China). No additional moisture measurements were performed for cotton samples.

### 2.4. Light Reflectance and Color Coordinate Measurements

For non-irradiated and UVC-irradiated cotton—NBT samples, the light reflectance in the wavelength range of 400–700 nm was measured, and the color coordinates were determined using a Spectraflash 300 reflectance spectrophotometer (DataColor, Rotkreuz, Switzerland). The measurements were performed with the microMATCH v.3.6 software (DataColor, Rotkreuz, Switzerland) with standard D65 illumination (angle of 10°; resolution of 10 nm; measurement error of 0.1%) according to a calibration procedure described elsewhere [12]. To limit the influence of the weave structure on the color changes of the samples during reflectance measurements, an average of five measurement samples was used. The cotton—NBT samples were also characterized in terms of the changes in the CIE L*, a*, and b* color coordinates, which were determined using the CIE Lab 1976 rating system, where “L*” is the lightness of the color (black to white on a scale of 0 to 100), the “a*” value is the color component on the green–red axis, and the “b*” value is the color component on the blue–yellow axis, in accordance with the ISO/CIE 11664-4 standard [13].

For selected samples, photographs were also taken using a Canon 50D digital camera (Canon Inc., Tokyo, Japan) in standard D65 lighting (color temperature 6504 K, Veri Vide F18T8/D65, Enderby, Leicester, Great Britain) with the kit lens EF-S set 18–200 mm f/3.5–5.6 with the following settings: resolution: 15.1 MPix; pixel size: 4.69 µm; without flash.

### 2.5. Two-Dimensional Dose Distribution Measurements

Cotton—NBT dosimeters (9 × 11 cm^2^) and calibration samples (2.5 × 2.5 cm^2^) after non-homogeneous UVC irradiation in the dose range 0–10,000 mJ/cm^2^ were scanned using an HP Scanjet G3010 flatbed scanner (Hewlett-Packard Company, Palo Alto, CA, USA). The following settings were used: resolution: 75 dpi; non-color corrections; and image sharpening. All cotton—NBT samples were scanned approximately 2 h after irradiation. The obtained bitmaps after scanning the cotton—NBT dosimeters were processed using the polyGeVero^®^-CT software package (v.1.2, GeVero Co., Lodz, Poland). After applying the calibration equation, the images were converted into 2D dose distribution images. Then, the dose maps were exported to the polyGeVero^®^ software package (v. 2.0, GeVero Co., Lodz, Poland) for further analysis. The following steps were performed using the software package: (i) filtering the images after scanning the cotton—NBT dosimeter; (ii) calibrating the dosimeter; (iii) converting the obtained results to the UVC dose distribution; and (iv) creating 2D maps with the UVC dose distribution. A detailed description of image processing with the polyGeVero^®^ and polyGeVero^®^-CT software packages is given elsewhere [9,10].

## 3. Results and Discussion

### 3.1. General Characteristics of the Cotton—NBT Dosimeters

**Figure 1:**
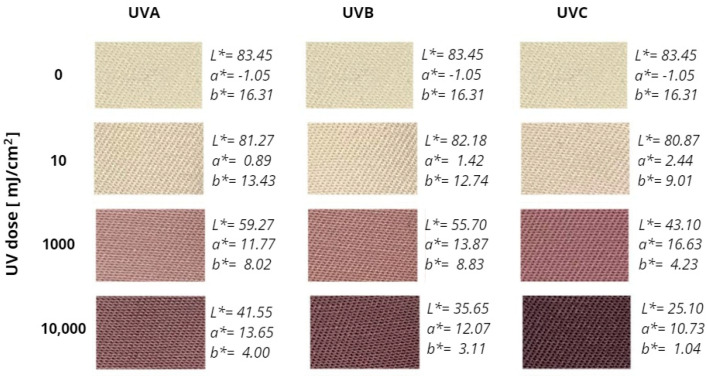
Example photographs of cotton—NBT dosimeters and their CIE L*, a*, and b* color coordinate values after irradiation with UVA, UVB, and UVC radiation in the dose range of 0–10,000 mJ/cm^2^.

Dosimeters based on woven fabrics modified with radiation-sensitive compounds, including tetrazolium salts and leuco-dyes, are an original idea from the DosLab research group, which has been described in the scientific literature for over fifteen years. Dosimetric systems manufactured by the screen printing and padding—squeezing—drying method using NBT were characterized in terms of their response to UV and ionizing radiation doses. Some of the developed solutions were also applied to measure the UV and ionizing radiation 2D dose distributions using the polyGeVero^®^-CT software package (v.1.2, GeVero Co., Lodz, Poland). The mechanism of the transformation of the NBT molecule into formazan is also known and has been described under different conditions in other publications [9,10,14]. Regardless of the type of radiation and the dosimeter manufacturing method, NBT-modified fabrics change color from light yellow to purple-brown. The intensity and rate of the color change depend on (i) the type of radiation, (ii) the range of radiation doses, (iii) the concentration of NBT used for modification, and (iv) the type of textile substrate, including the weave structure. In general, the higher the concentration of NBT, the more pronounced the response of the samples to radiation is. In the case of UVA (315–400 nm), UVB (280–360 nm), and UVC (100–280 nm) radiation, a similar nature of color change was observed in the cotton—NBT dosimeters after irradiation, but in the case of UVC, the intensity of color changes was the most pronounced. Comparing the CIE L*, a*, and b* values for individual doses, it was found that the UVC-irradiated samples achieved a higher degree of color conversion. Photographs of the selected cotton—NBT dosimeters and changes in the CIE L*, a*, and b* color coordinates values after irradiation with UVA, UVB, and UVC radiation in the range of 0–10,000 mJ/cm^2^ are presented in Figure 1.

Directly after the preparation of the cotton—NBT dosimeter, all samples were light yellow with a visible fabric weave structure. In the case of textile dosimeters, the structure of the textile material plays an important role in the process of reading information about the dose and the 2D distribution of the radiation dose. The modified textile material must maintain its size and shape during dosimeter preparation, irradiation, and measurements. Therefore, knitted and nonwoven fabrics, which are more stretchable, were not used to construct textile dosimeters. In previous work, the influence of fabric structure on 2D measurements of radiation dose was also investigated using a flatbed scanner. It was shown that fabrics with simple, plain weaves are more suitable for the construction of dosimeters due to better surface uniformity and less visibility of the weave after sample irradiation. Moreover, if the padding—squeezing—drying method is used for textile dosimeter manufacturing, it is suitable to use cotton fabric due to the even absorption of the solution with the radiation-sensitive compound by hydrophilic cellulose fibers. It should be emphasized that access to white cotton fabrics, with a plain weave and without additional refining agents, is very limited. Typically, these types of fabrics are additionally finished with optical brighteners, which adversely affect the uniformity of color changes in the dosimeter after irradiation and cause errors during light reflectance measurements and the determination of the CIE L*, a*, and b* color coordinates. In addition, our limited access to fabric manufacturers did not allow for the production of a model fabric with the specified technological parameters. Therefore, in the presented research, a white cotton fabric with a twill weave was used, and only UVC radiation was used for sample irradiation.

### 3.2. Repeatability of Reflectance Measurements vs. Storage Conditions

**Figure 2:**
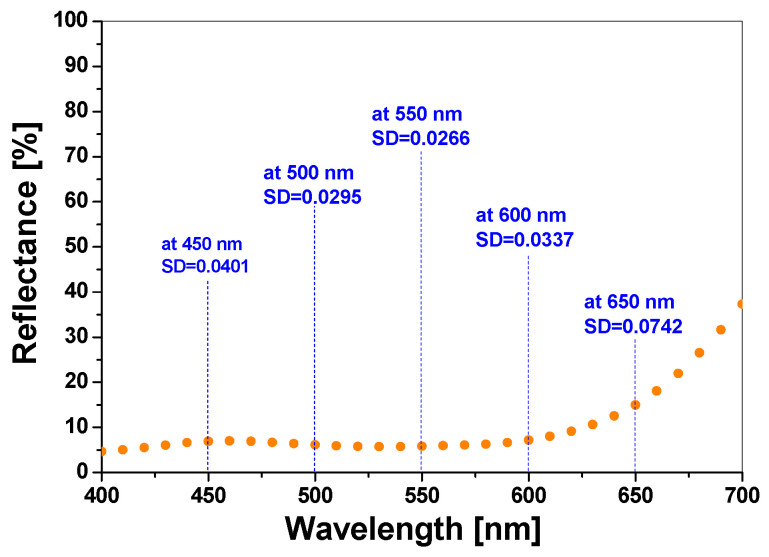
Spectrum of the light reflectance (orange dots) as a function of the wavelength in the range of 400–700 nm for measurements of 15 cotton—NBT samples after irradiation with a dose of 10,000 mJ/cm^2^ UVC.

To investigate the repeatability of light reflectance measurements and the reproducibility of samples, 15 cotton-NBT samples were prepared according to the procedure described in Section 2.1 and irradiated with a dose of 10,000 mJ/cm^2^ UVC. High-dose UVC irradiation induced maximum conversion of the radiation-sensitive compound and a color change that can be obtained for a given NBT concentration. Thus, during the reflectance measurements, it was not necessary to use additional shields to avoid accidentally exposing the samples to radiation, e.g., from artificial light sources from lamps illuminating the measurement room. For all cotton—NBT samples, the light reflectance in the wavelength range of 400–700 nm was measured with a Spectraflash 300 reflectance spectrophotometer under standard D65 illumination (angle of 10°; resolution of 10 nm; measurement error of 0.1%). To limit the influence of the weave structure on the color changes of the samples during reflectance measurements, an average of five measurement samples was used. An equation characterizing the repeatability of the obtained measurements at 550 nm was also determined, Reflectance (λ = 550 nm) [%] = 5.8702 [%]−0.0046 [%] × Sample number [−], R^2^ = 0.8647, and the results are presented in Figure 2. For example, the standard deviation (SD) was 0.0401, 0.0295, 0.0266, 0.0337, and 0.0742 at wavelengths of 450, 500, 550, 600, and 650 nm, respectively.

**Figure 3:**
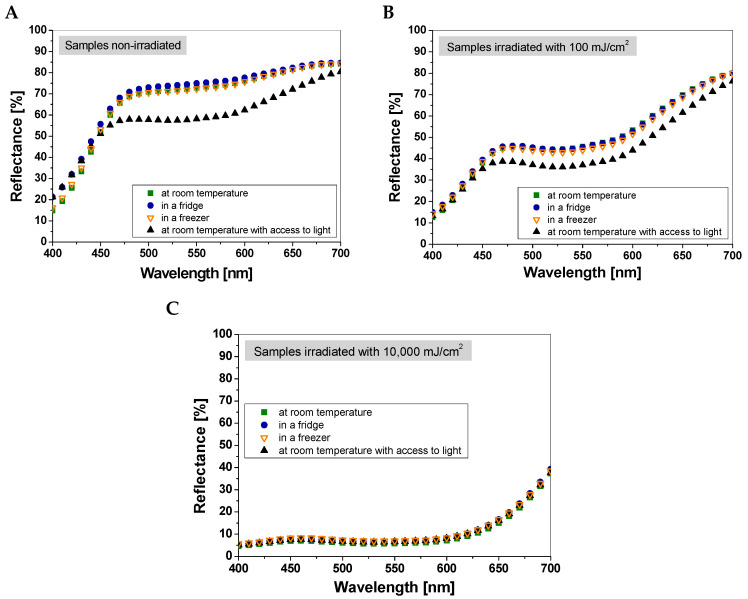
Evaluation of measurement repeatability of cotton—NBT dosimeters stored under different conditions for non-irradiated samples (**A**), samples irradiated with a dose of 100 mJ/cm^2^ (**B**), and 10,000 mJ/cm^2^ (**C**) after 24h from their preparation. The graphs show the average results from measurements of five samples for each storage variant.

Thus, it was concluded that textile dosimeters made from a single fabric batch have satisfactory reproducibility. Therefore, the average measurements from five samples of cotton—NBT at 550 nm were used in the studies on sample storage conditions. In the next step, directly after preparation, the cotton—NBT samples were divided into four series, which were stored in different conditions: (i) at room temperature (23–25 °C, humidity 40–60%, without access to light); (ii) in a fridge (3–5 °C, humidity 70–90%, without access to light); (iii) in a freezer (−17 to −20 °C, humidity 80–90%, without access to light); and (iv) at room temperature (23–25 °C, humidity 40–60%), with access to light from lamps illuminating the laboratory room. Temperature changes in the laboratory room, fridge, and freezer may result from (i) closing and opening the fridge and freezer, (ii) temperature fluctuations in the laboratory room during the day, and (iii) seasonal changes in the laboratory room during the experiment over a six-month period from summer to winter. It is important that the first light reflectance measurement in all series was performed after 24 h of the samples being in the given temperature and humidity conditions. For the storage of the dosimeters in the fridge and freezer, the cotton—NBT samples were taken out two hours before UVC irradiation to reach ambient temperature and eliminate water vapor condensation on the sample surface. Measurements of cold or frozen samples may cause unwanted water vapor condensation on the fabric surface and contribute to increased measurement uncertainty. Furthermore, according to the ISO 105-J01:1997 [15] standard, the color of textiles should be assessed for dry fabric at ambient temperature, without additional conditioning and ironing. No additional moisture measurements were performed for the cotton—NBT samples. To analyze the measurement repeatability of dosimeters stored under different conditions (the similarity and differences between the samples with respect to their color), light reflectance measurements were performed for non-irradiated and irradiated samples with a dose of 100 and 10,000 mJ/cm^2^ UVC after 24 h from their preparation. Five samples were selected from each series, and after averaging the measurement points, the results are presented in Figure 3.

In the case of non-irradiated and 100 mJ cm^2^-irradiated samples (Figure 3A,B), a similarity in the spectral curves can be seen. In both cases, the largest change in light reflectance was visible for the sample stored at room temperature with access to light. For non-irradiated cotton—NBT dosimeters, the differences in light reflectance at 550 nm between the samples stored at room temperature, in a fridge, in a freezer, and at room temperature with access to light were 2.78%, 0.68%, and 22.34%, respectively. For samples irradiated with 100 mJ/cm^2^, the difference between the reflectance for samples (in the same storage conditions) was 0.48%, 3.63%, and 18.45%, respectively. For cotton—NBT samples irradiated with a dose of 10,000 mJ/cm^2^ UVC, the change in light reflectance depending on storage conditions is not so pronounced (Figure 3C). Comparing the light reflectance at 550 nm values, the differences between the sample stored at room temperature and those stored in the fridge, freezer, and room temperature with access to light were 0.17%, 0.67%, and 0.53%, respectively.

### 3.3. Long-Term Stability and Dose Response of Cotton—NBT Dosimeters

**Figure 4:**
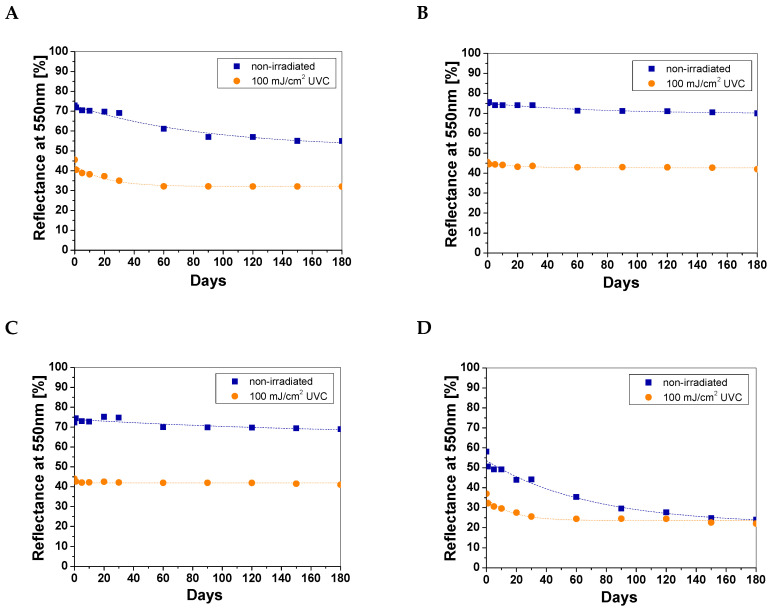
Stability measurements over time for non-irradiated and irradiated cotton—NBT samples irradiated with a dose of 100 mJ/cm^2^ UVC and stored for 180 days at room temperature (**A**); in a fridge (**B**); in a freezer (**C**); and at room temperature with access to light (**D**).

The stability over time of the cotton—NBT dosimeters was determined over a period of 180 days from their preparation. Based on previous work [8,9] and experience with cotton—NBT dosimeters, the results were analyzed for reflectance at 550 nm and are presented in Figure 4. As in the case of sample repeatability measurements, each point on the graph is the average value of five measurement points. Comparing the obtained light reflectance results, the highest stability over time was observed for the cotton—NBT samples stored in the fridge and freezer (Figure 4B,C). In the case of samples stored at room temperature, the non-irradiated samples change color much faster. After 180 days, the shade difference was 23.93% and 54.19% for the samples stored at room temperature under an aluminum foil cover and at room temperature with access to light, respectively (Figure 4A,D).

**Figure 5:**
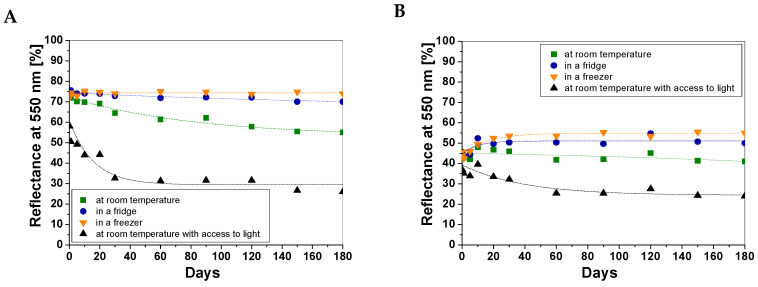
Comparison of the impact of time and storage conditions on the color intensity changes of non-irradiated cotton—NBT samples (**A**) and their dose response after 100 mJ/cm^2^ of UVC irradiation (**B**). Each measurement point on the graphs is the average value of three measurements taken for the same sample.

Moreover, the color change under these conditions for the 100 mJ/cm^2^ UVC-irradiated cotton—NBT samples was also more intensive. During the initial period of storage of the samples at room temperature, the light remission value decreased by 8.03% and 23.01% for the samples stored at room temperature under an aluminum foil cover and at room temperature with access to light, respectively. The characteristics of the changes occurring during sample storage are presented in Table 1. The percentage change in color intensity was calculated by comparing the reflectance values for a given sample with a blank sample within 180 days of dosimeter preparation. Thus, it was concluded that non-irradiated cotton—NBT samples should be stored in a freezer or fridge because, under these storage conditions, the change in color intensity was the smallest. However, in the case of the UVC-irradiated samples, storage at room temperature with a cover of aluminum foil turned out to be better. It should be emphasized that, in practice, dosimeters of the single-use type are very rarely stored over long periods of time. They cannot be stored in direct contact with non-irradiated samples to limit possible post-radiation reactions between dosimeters and their influence on dose measurement and uncertainty. If there are no special procedures for their deactivation or disposal, they are simply thrown away. However, in the case discussed above, the greatest influence on the color changes of the cotton—NBT dosimeters was probably the change in the humidity of the samples. Thus, in the next stage of the study, the influence of storage time and conditions on the response of the cotton—NBT dosimeter to the UVC dose was analyzed. For this purpose, 12 samples were irradiated with a dose of 100 mJ/cm^2^ at different time intervals to demonstrate the repeatability of the dose response and thus the repeatability of production and to determine the optimal shelf life (Figure 5).

For non-irradiated cotton—NBT samples, the obtained results confirmed that storage in a freezer and fridge is the most optimal due to the high stability of the color intensity for 180 days (Figure 5A). After irradiation of the same samples with a dose of 100 mJ/cm^2^, a slight decrease in the color intensity of the samples (higher reflectance values) was observed for up to 20 days (Figure 5B). This effect was also visible for samples stored at room temperature. Only for samples stored at room temperature with access to light it was visible that their color intensity increased over 20 days of storage (decrease in reflectance values). For example, the change in light reflectance values over 20 days from the preparation of the non-irradiated cotton—NBT dosimeter was 5.21%, 2.43%, 39.36%, and 3.88% for samples stored at room temperature, in a fridge, in a freezer, and at room temperature with access to light, respectively. Of course, cotton—NBT dosimeters can be used immediately after preparation. Depending on the type of application and the nature of changes in dosimeters during storage, it seems that the optimal solution would be to start using cotton—NBT dosimeters for dose measurements at least 20 days after their preparation. For example, the difference in color of the samples over 20–180 days did not exceed 2.72% and 4.81% for samples stored in a fridge and freezer, respectively. However, the scatter of points may also result from the fact that the measurements of textile products are difficult due to their texture, and therefore, not only the weave but also the accidental creasing of the sample resulting from touching it may affect the light reflectance measurement.

### 3.4. Radiation 2D Dose Distribution vs. Storage Conditions of the Cotton—NBT Dosimeter

**Figure 6:**
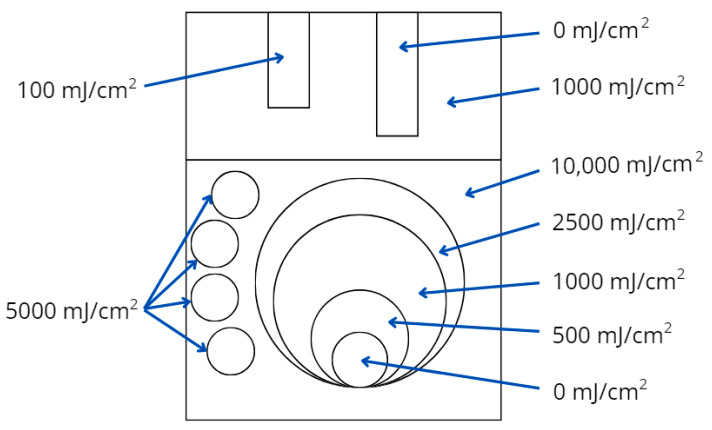
Scheme of non-homogeneously irradiated cotton—NBT dosimeters in the dose range of 0–10,000 mJ/cm^2^.

**Figure 7:**
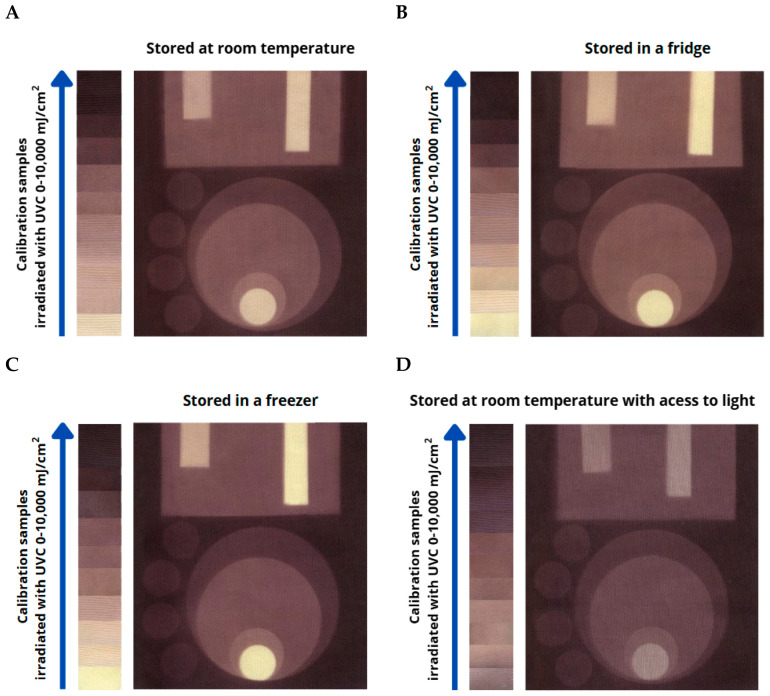
Comparison of color change effects after non-homogeneous UVC irradiation of cotton—NBT samples stored under different conditions for eight months from their preparation. Samples were stored at room temperature (**A**), in a fridge (**B**), in a freezer (**C**), and at room temperature with access to light (**D**). The blue arrow indicates the increase in color intensity of the calibration samples as a result of UVC irradiation.

The cotton-NBT dosimeter samples, stored under different conditions described in previous sections, were also non-homogeneously irradiated to confirm that we could register a 2D UV dose distribution eight months after their preparation. To perform non-homogeneous UVC irradiation, brass metal elements were placed on the surface of the samples. It was important that during irradiation, these objects adhered well to the fabric surface, did not reflect UV radiation, and did not create additional artifacts on the textile substrate. Cotton—NBT dosimeters with calibration samples were irradiated with UVC doses in the dose range of 0–10,000 mJ/cm^2^. The scheme of non-homogeneous UVC irradiation is presented in Figure 6. After irradiation, all samples were scanned with a flatbed scanner, and the obtained images of the cotton—NBT samples after scanning are presented in Figure 7. Regardless of the storage conditions, all cotton—NBT samples showed a visible fabric weave structure after scanning. Previous research has shown that the quality of images obtained after scanning textile dosimeters is influenced by (i) the type of textile substrate, including its weave, used for the dosimeter preparation; (ii) the scanning resolution and scanner control software settings; and (iii) the averaging of data after scanning [9,10,16]. In addition, the samples after irradiation looked very similar to each other. Of course, the difference in shade was due to the storage conditions of the cotton—NBT samples over eight months, but after irradiation, the samples changed color from yellow to purple-brown, which indicates the possibility of using such dosimeters for longer periods of time. However, the samples stored at room temperature (Figure 7A,B) were slightly darker than those stored in the fridge and freezer (Figure 7B,C).

**Figure 8:**
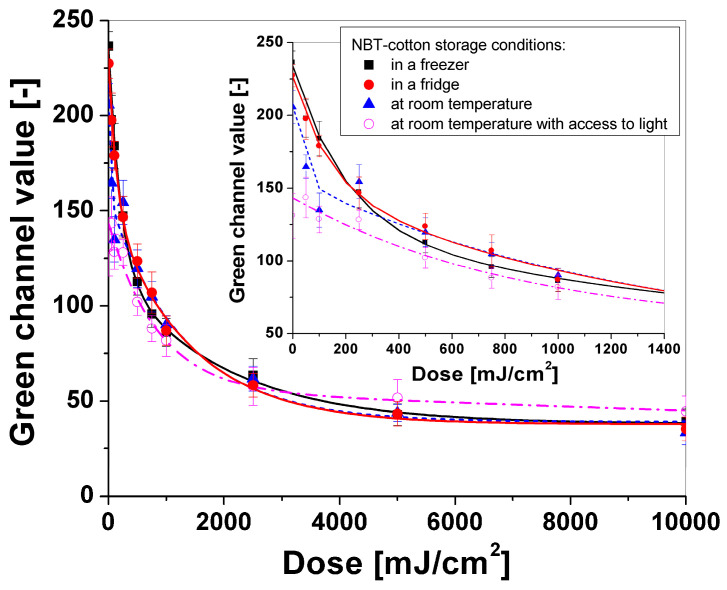
Calibration relations for cotton-NBT dosimeter storage under different conditions for eight months before UVC irradiation.

After irradiation, the samples were scanned using a flatbed scanner at a 75 dpi resolution without additional color correction and image sharpening. The use of low-resolution scanning allows for a reduction in noise from the weave of the textile substrate, which affects the quality of images after applying the filtering described in Section 2.5. The images of the cotton–NBT calibration samples after scanning were then processed using the polyGeVero^®^-CT software package to decompose them into color channels in the RGB color model. All RGB channels contributed significantly to the color change in the irradiated cotton—NBT samples, similarly to previous research [9,10]. However, the greatest change in color intensity was visible for the green channel, which was selected for further analysis. Using the tools of the polyGeVero^®^ software package, all images were filtered. The following settings were used: average filter; kernel size: 3; kernel unit: mm; kernel mode: 2D; iterations: 2. The selected settings allowed for smoothing the images of the scanned cotton—NBT samples without distorting their dimensions and shapes. This procedure allowed for the preparation of a calibration relation between the green channel values and the UVC-absorbed dose, which is presented in Figure 8. Each measurement point is the average value of the entire calibration sample image, with an area of 7000–10,000 points and the standard deviation bars marked. After analyzing the dose response of the cotton—NBT dosimeters, it can be concluded that regardless of the storage conditions, the dynamic dose response reaches up to approximately 2500 mJ/cm^2^. Above a dose of 3000 mJ/cm^2^, a slight change in the dose response of the dosimeters to the UVC was observed. It was also seen that the dose sensitivity decreased with increasing purple-brown color conversion, becoming apparent in the dose range above 100 mJ/cm^2^. The decrease in the green channel value as a function of the absorbed dose can be described by the following calibration equation: Green channel = A1 × exp(-Dose/t1) + A2 × exp(-Dose/t2) + y0. Table 2 presents the characteristics of dosimeters stored in the freezer, in the fridge, at room temperature, and at room temperature with access to light.

**Figure 9:**
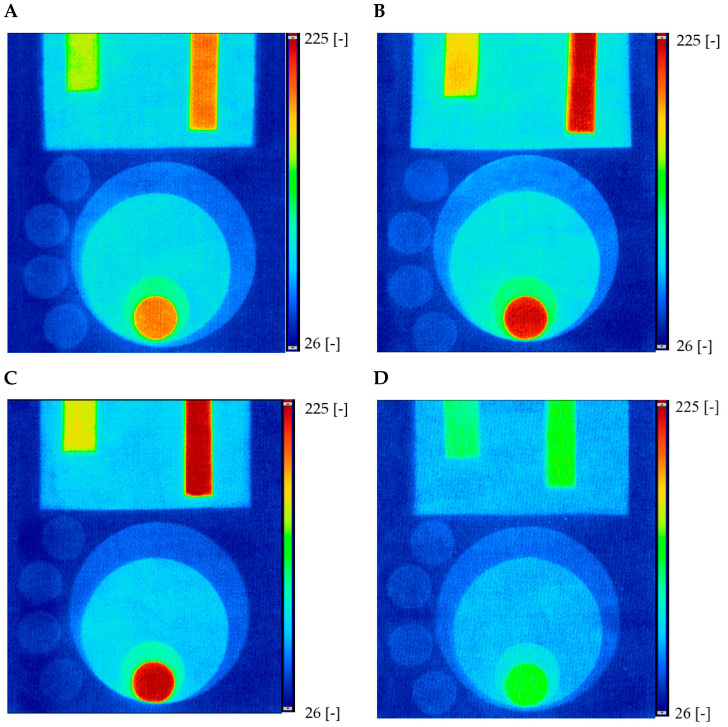
Comparison of green RGB channels for cotton—NBT dosimeters after exposure to UVC inhomogeneous irradiation for samples stored in different conditions: at room temperature (**A**), in a fridge (**B**), in a freezer (**C**), and at room temperature with access to light (**D**). The color scale (from 26 to 255) determines the changes in color intensity in the green RGB channel.

For non-homogeneously irradiated samples, the same procedure was followed. After scanning, the obtained images were processed with the polyGeVero^®^ software package, and the obtained images in the green RGB channel are presented in Figure 9. The results confirm that the greatest change in color intensity was observed in samples stored in the freezer and fridge (Figure 9B,C). In the case of the samples stored at room temperature, a decrease in color intensity was observed in the green channel, but it was still clearly visible.

**Figure 10:**
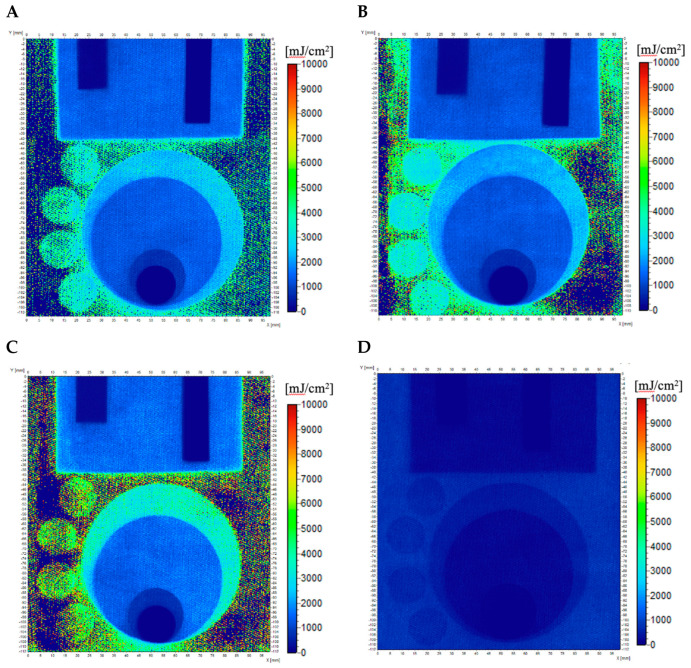
Dose distribution maps calculated using calibration equations from Table 2 for cotton—NBT dosimeters after exposure to UVC inhomogeneous irradiation for samples stored in different conditions: at room temperature (**A**), in a fridge (**B**), in a freezer (**C**), and at room temperature with access to light (**D**).

After applying the calibration equations described previously, 2D UVC dose distribution maps were also generated and are presented in Figure 10. Comparing the results obtained from the 2D distribution maps with the dose emitted by the UV source, after applying the calibration equation and assigning a color scale, the samples stored in the fridge and freezer registered a maximum dose of around 8000 mJ/cm^2^ (Figure 10B,C). In both cases, the sample surface shows marked red areas indicating the registration of higher doses, but they are random and not limited to the shape irradiated with 10,000 mJ/cm^2^. It is assumed that this noise is related to the weave of the fabric, which appears darker in these areas. Therefore, after applying the calibration equation, the color scale suggests that these are areas irradiated with a higher UVC dose. For the sample stored at room temperature (Figure 10A), the maximum registered dose is about 5000 mJ/cm^2^, and for the sample stored with access to light (Figure 10D), it is 1500 mJ/cm^2^. For this last sample, the changes after applying the calibration equation are practically invisible in the obtained image. This is due to the sample’s lower sensitivity to the dose and its lower reflectance value before irradiation, which occurred after it was stored at room temperature for eight months without a foil cover and access to light.

In summary, the difference between the emitted and absorbed doses is visible and may be due to the following reasons: (i) the change in the sensitivity of the dosimetry system during the eight months of storage; (ii) partial scattering of UVC radiation on the elements covering the sample during non-homogeneous irradiation; (iii) the structure of the textile substrate used to prepare the dosimeter; (iv) the selection of parameters used during filtering and image processing after scanning; and (v) the calibration of the dosimetry system. However, the conducted studies prove that the cotton—NBT dosimeters are suitable for dose measurements and 2D dose distribution of UVC radiation with a non-homogeneous pattern eight months after dosimeter preparation.

## 4. Conclusions

The presented work showed that the dosimeter based on cotton fabric and an NBT aqueous solution, prepared by the padding—squeezing—drying method, can be used for UVC radiation dose measurements and obtaining the two-dimensional dose distribution if stored in controlled conditions, preferably in a freezer or fridge, for up to eight months after its production. The cotton—NBT dosimeter is easy to prepare and can be produced in large batches using standard machinery used in the textile industry. Optimizing conditions for the production, storage, and use of textile dosimeters can increase their availability and reduce the production costs of this type of system for radiation field control and UVC radiation measurements. Storage conditions influence the effectiveness of textile dosimeters over time. It is important to ensure an appropriate temperature during their storage, below 5 °C, and to limit access to light by covering the dosimeters, e.g., with aluminum foil. However, the biggest problem is the spontaneous color change during the first 20 days after the preparation of the dosimeters and their conditioning after taking them out of the freezer and fridge. By scanning and filtering images using computer software, comparative measurements and professional dose assessment in real time are possible. Therefore, cotton—NBT dosimeters can be used as a method complementing standardized processes for the calibration and control of UVC radiation measurement systems. The obtained results do not fully solve the issues related to the stability and storage conditions of textile dosimeters. Future studies should focus on the following: (i) the influence of humidity on the stability of dosimeters over time; (ii) the influence of packaging type and access to oxygen on color changes during the storage of dosimeters; (iii) the selection of a sample conditioning method after removal from the fridge or freezer; and (iv) the selection of software settings during image filtering to better match the calibration relation with the absorbed dose.

## Figures and Tables

**Table 1:** The percentage change in light reflectance values at 550 nm for non-irradiated and irradiated cotton—NBT samples within 180 days of their preparation under different storage conditions.

**Non-Irradiated Samples**					
**Days**	**1**	**5**	**10**	**30**	**180**
At room temperature with access to light	13.10%	15.46%	24.30%	43.79%	54.19%
At room temperature	1.27%	3.66%	4.17%	11.39%	23.93%
In a fridge	0.62%	1.43%	1.56%	3.01%	6.72%
In a freezer	0.88%	2.96%	3.66%	4.18%	4.25%
**UVC-Irradiated (100 mJ/cm^2^) Samples**					
**Days**	**1**	**5**	**10**	**30**	**180**
At room temperature with access to light	4.70%	8.46%	11.25%	23.01%	34.33%
At room temperature	5.88%	7.48%	7.76%	8.03%	9.17%
In a fridge	2.35%	3.25%	5.78%	8.93%	11.44%
In a freezer	2.76%	4.85%	7.30%	16.50%	26.57%

**Table 2:** Calibration function parameters for cotton-NBT dosimeter samples kept under different conditions for eight months: in a freezer (no. 1), in a fridge (no. 2), at room temperature (no. 3), and at room temperature with access to light (no. 4). The following function was used: Green channel = A1 × exp(−Dose/t1) + A2 × exp(−Dose/t2) + y0.

No.	A1	t1	A2	t2	y0	R^2^
1	82.601 ± 8.873	1926.852 ± 446.196	113.455 ± 10.132	203.260 ± 32.516	37.950± 3.208	0.997
2	114.779 ± 10.817	1379.437 ± 225.900	74.081 ± 11.870	134.955 ± 33.906	37.794 ± 2.823	0.996
3	116.814 ± 13.044	1320.777 ± 306.617	50.375 ± 18.601	31.783 ± 27.595	39.043 ± 4.932	0.974
4	3.571 × 10^6^ ± 1.759 × 10^8^	3.424 × 10^9^ ± 1.687 × 10^11^	87.935 ± 11.137	851.786 ± 207.766	−3.571 × 10^6^ ± 1.759 × 10^8^	0.965

## Data Availability

The original contributions presented in the study are included in the article; further inquiries can be directed to the corresponding author/s.
